# Proteomic and functional annotation analysis of injured peripheral nerves reveals ApoE as a protein upregulated by injury that is modulated by metformin treatment

**DOI:** 10.1186/1744-8069-9-14

**Published:** 2013-03-26

**Authors:** Ohannes K Melemedjian, Hussein N Yassine, Adia Shy, Theodore J Price

**Affiliations:** 1 Department of Pharmacology, The University of Arizona School of Medicine, 1501 N Campbell Ave, Tucson, AZ, 85724, USA; 2 Department of Medicine, The University of Arizona School of Medicine, Tucson, AZ, USA; 3 Bio5 Institute, The University of Arizona, Tucson, AZ, USA; 4 Graduate Interdisciplinary Program in Neuroscience, The University of Arizona, Tucson, AZ, USA; 5 Department of Medicine, The University of Southern California, Los Angeles, CA, USA

## Abstract

**Background:**

Peripheral nerve injury (PNI) results in a fundamental reorganization of the translational machinery in the injured peripheral nerve such that protein synthesis is increased in a manner linked to enhanced mTOR and ERK activity. We have shown that metformin treatment, which activates adenosine monophosphate-activated protein kinase (AMPK), reverses tactile allodynia and enhanced translation following PNI. To gain a better understanding of how PNI changes the proteome of the sciatic nerve and ascertain how metformin treatment may cause further change, we conducted a range of unbiased proteomic studies followed by biochemical experiments to confirm key results.

**Results:**

We used multidimensional protein identification technology (MUDPIT) on sciatic nerve samples taken from rats with sham surgery, spinal nerve ligation (SNL) surgery or SNL + 200 mg/kg metformin treatment. MUDPIT analysis on these complex samples yielded a wide variety of proteins that were sorted according to their peptide counts in SNL and SNL + metformin compared to sham. These proteins were then submitted to functional annotation analysis to identify potential functional networks altered by SNL and SNL + metformin treatment. Additionally, we used click-chemistry-based labeling and purification of nascently synthesized proteins followed by MUDPIT to further identify peptides that were synthesized within the injured nerve. With these methods, we identified apolipoprotein E (ApoE) as a protein profoundly increased by PNI and further increased by PNI and metformin. This result was confirmed by Western Blot of samples from SNL rats and spared nerve injury (SNI) mice. Furthermore, we show that 7-day treatment with metformin in naïve mice leads to an increase in ApoE expression in the sciatic nerve.

**Conclusions:**

These proteomic findings support the hypothesis that PNI leads to a fundamental reorganization of gene expression within the injured nerve. Our data identify a key association of ApoE with PNI that is regulated by metformin treatment. We conclude from the known functions of ApoE in the nervous system that ApoE may be an intrinsic factor linked to nerve regeneration after PNI, an effect that is further enhanced by metformin treatment.

## Background

Despite advances in our understanding of the pathogenesis of neuropathic pain, the clinical treatment of this disorder remains problematic [1]. Hence, there is an urgent need for novel treatment approaches targeting molecular mechanisms of pathology induced by peripheral nerve injury (PNI). Injured neurons from mammalian peripheral or invertebrate central nerves retain a capacity for functional regeneration. This regeneration process apparently involves a re-organization of the translational capacity of injured axons [2-4]. The change in axonal protein synthesis after PNI may be functionally linked to the development and persistence of neuropathic pain [5-8]. Using rationale pathway analysis approaches we have shown along with others that neuropathic pain is correlated with enhanced mammalian target of rapamycin complex 1 (mTORC1) [5,7] and extracellular signal regulated protein kinase (ERK) [7] activity and that blocking these pathways attenuates some sequelae of neuropathic pain [5-8]. An important piece of evidence linking these pathways to neuropathic pain is the finding that pharmacologically stimulating adenosine monophosphate-activated protein kinase (AMPK), which inhibits signaling through these pathways [9], leads to a resolution of neuropathic allodynia [7].

While these findings link mTOR and ERK activity in peripheral neurons following PNI to neuropathic pain, downstream effectors generated via enhanced translation in the axonal compartment have not been identified. However, several previous studies have suggested candidate genes. First, a proteomic profiling of neuromas generated by PNI suggested an important role for translation control pathways in neuroma-generated pain [10]. Second, an important contributor to neuropathic [11-14] and other types of pathological pain [15-17], voltage-gated Sodium channel type 1.8 (Na_v_1.8) mRNA, localizes to the axonal compartment. This suggests that this crucial channel can be translated on-demand in the axons of nociceptors [18]. There is also some evidence that TRPV1 mRNA localizes to axons [19,20] suggesting it can be synthesized in the axonal compartment of nociceptors. While the candidate protein or single pathway approach can be successful, the anatomical and molecular complexity of the nervous system suggest that most signaling systems are not likely to be critically dependent on single pathways. This appears to be the case in terms of changes in translation control after nerve injury and is a major part of the underlying rationale to engage AMPK as a potential treatment for neuropathic pain [7].

It is also clear that changes in gene expression in cells, other than neurons, either intrinsic to or invading the injured peripheral nerve may play a crucial role in neuropathic pain and/or functional recovery after PNI [21]. In this regard, a variety of extrinsic cell types have been identified [22] and the activation of other, intrinsic cells has been positively correlated with pain induced by PNI [23]. While it is clear that changes in translation regulation occur in the axonal compartment after PNI [7], it is undoubtedly also true that changes in these pathways may contribute to neuropathic pain and that AMPK activation may be physiologically linked to these pathways in such non-neuronal cells.

To gain a better understanding of how PNI changes the proteome of the sciatic nerve, including axons, intrinsic and extrinsic cells, and to ascertain how stimulation of AMPK with metformin treatment may cause further change, we conducted a range of unbiased proteomic and bioinformatic studies on the sciatic nerve of rats with or without PNI including PNI and metformin treatment. Our findings suggest a complex change in PNS signaling networks associated with nerve injury and its further modulation via treatment with metformin. Moreover, our results, confirmed with biochemical studies, demonstrate that apolipoprotein E (ApoE) is strongly upregulated by PNI and nascently synthesized in the injured sciatic nerve. Interestingly, ApoE, which is linked to neuroprotection in neurodegenerative and traumatic nerve injury, is further increased by metformin treatment suggesting a potential role of ApoE in functional recovery and/or reduction of neuropathic pain after PNI. Overall, our results create a framework for the better understanding of changes in translation control that occur following nerve injury and their potential contribution to neuropathic pain and nerve regeneration.

## Results

We first sought an unbiased view of the proteome of the sciatic nerve following proximal injury to the L5 and L6 spinal nerves using the SNL model in rats. We also investigated how metformin treatment alters the injured proteome by giving rats I.P. injections of 200 mg/kg metformin daily for 7 days commencing 14 days following SNL surgery. Sciatic nerve samples were taken 21 days following SNL and analyzed by MUDPIT after pooling samples from 6 animals per group. A control group received sham surgery and vehicle treatments, while the SNL group without metformin also received vehicle injections. With MUDPIT we identified a total of 295 proteins with assigned peptides from all samples (Additional file 1: Table S1). Using these assigned peptides, we subdivided proteins into groups based on the number of peptides observed in sham, SNL and SNL + metformin groups for each protein by MUDPIT analysis. Seventy-eight proteins were identified where a higher absolute number of peptides were observed in the sciatic nerve compared to sham animals (Additional file 1: Table S2). One of the most increased peptides was ApoE (82 injured vs. 0 sham). In contrast, 43 proteins were identified where the absolute number of peptides was lower in the injured sciatic nerve than in the sham sciatic nerve (Additional file 1: Table S3). We also examined proteins that had increased peptide counts with metformin + SNL compared to SNL alone. Here we found 66 proteins (Additional file 1: Table S4), including ApoE (290 metformin + SNL vs. 82 SNL peptides). Seventy-three proteins showed decreased peptide counts in the metformin + SNL group vs. the SNL group (Additional file 1: Table S5).

**Figure 1:**
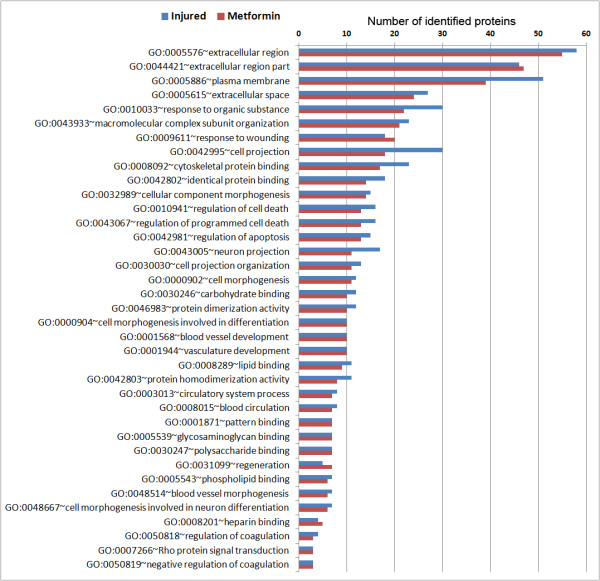
**Functional annotation data derived from DAVID algorithm (Part One).** Graph derived from the functional annotation chart that shows all cellular functions that include ApoE as a component (ApoE is assigned to that functional annotation) given as the number of proteins identified by MUDPIT with that specified functional annotation. The numbers indicate the number of proteins detected in the proteomic study that are clustered in that functional annotation (all of which include ApoE) following SNL (blue) or SNL with metformin (red) treatment. The graph includes functional annotations where the protein number in injured sciatic nerves (blue) and in injured nerves following metformin treatment (red) is not different. Hence, these functional annotations are not modulated by metformin and appear to be associated with an injury response that is resistant to metformin treatment.

**Figure 2:**
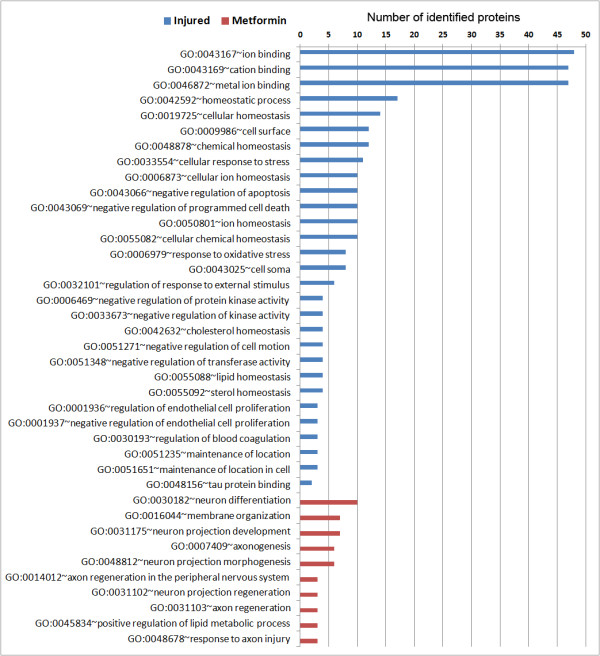
**Functional annotation data derived from DAVID algorithm (Part Two).** Graph derived from the functional annotation chart that shows all cellular functions that include ApoE as a component (ApoE is assigned to that functional annotation) given as the number of proteins identified by MUDPIT with that specified functional annotation. The numbers indicate the number of proteins detected in the proteomic study that are clustered in that functional annotation (all of which include ApoE) following SNL (blue) or SNL with metformin (red) treatment. The graph indicates number of proteins for functional annotations containing ApoE that are unique to injured nerves (blue) and are not apparent with metformin treatment (lack of red bars). Moreover, metformin treatment induces the expression of number of proteins that belong to functional annotations containing ApoE that are not observed with nerve injury. These functional annotations are linked to enhance regeneration and repair of the peripheral nervous system (red) and these functional annotation sets are absent in SNL rats (lack of blue bars).

Having identified proteins that were altered by SNL and SNL + metformin, we sought to identify functional properties of this dataset. To do this we utilized The Database for Annotation, Visualization and Integrated Discovery (DAVID). This allowed us to determine enriched cellular functions following nerve injury or metformin treatment combined with nerve injury. Within the total proteomic dataset, we identified 780 functional annotations in DAVID from proteins identified from those two groups (Additional file 2: Table S6). Individual datasets are shown in Additional file 2: Tables S7 and S8. We then constructed a graph to visualize this data by protein number per functional annotation (Additional file 2: Graph 1). We hypothesized that ApoE, which is a pleiotropic protein with a wide range of identified cellular and physiological functions (functional annotations that include ApoE as a component are highlighted in red in Additional file 2: Tables S7 and S8 and the complete list of 295 functional annotations is found in Additional file 3: Table S9), may reveal novel insight concerning potential mechanisms of action of metformin because this protein was strongly increased in SNL rats and further increased by metformin treatment. To test this, we looked at functional annotations after SNL or SNL + metformin that included ApoE as a component and looked at the number of other proteins identified in the proteome from those samples that matched those annotations. The complete list is shown in Additional file 3: Table S10 and is shown graphically in Figures 1 and 2. Interestingly, we noted several functional annotations related to development and repair that were enriched both in the SNL and SNL + metformin groups. We also noted several functional annotations that were unique to the SNL group and that were decreased following metformin treatment with SNL. Interestingly, these functional annotations were largely related to ionic and other forms of homeostasis that might be compensatory mechanisms related to the development of ectopic activity in injured nerves (Figure 2). Crucially, in the SNL + metformin group, there was a strong enrichment of functional annotations linked to regeneration, differentiation and repair of the peripheral nervous system that were unique to this treatment (Figure 2). Hence, using a completely unbiased proteomic screen and an unbiased functional annotation algorithm (DAVID), we have identified an enrichment of functional annotations linked to nerve injury induced regeneration and repair induced by metformin based on the identification of ApoE as a protein strongly induced by nerve injury and further increased by metformin. These functional annotations include “axon regeneration in the peripheral nervous system”, “axonogenesis”, “axon regeneration” and “response to axon injury” (Figure 2). These functional annotations also tended to include microtubule associated protein 1B (IPI00372009) and neurofilament light polypeptide (IPI00231302).

We then utilized click-chemistry-based labeling and purification of nascently synthesized proteins followed by MUDPIT to further identify peptides that were nascently synthesized within the injured nerve. This was done using an ex-vivo approach as detailed in methods. With this unbiased method, we identified 14 proteins, including ApoE (Additional file 4: Table S11). Hence, we show that in addition to being increased by SNL and further increased by metformin treatment, ApoE is nascently synthesized in the injured sciatic nerve (2 peptides in SNL vs. 0 in sham).

**Figure 3:**
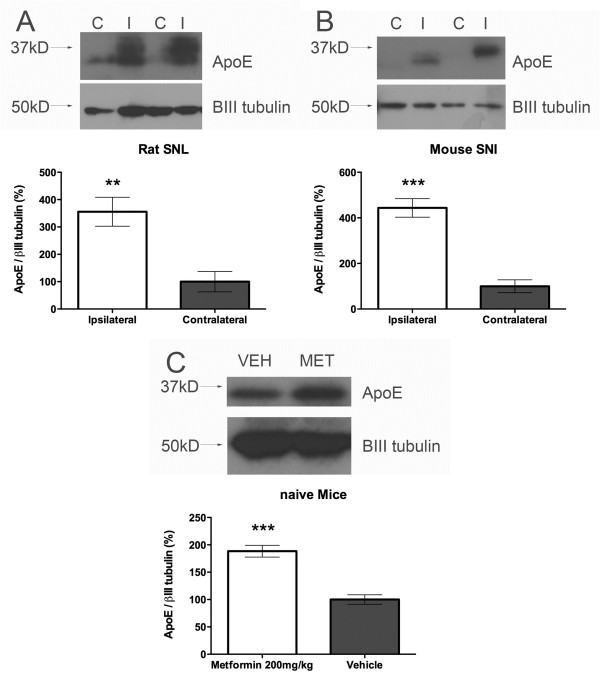
**ApoE expression is increased in the sciatic nerve following PNI and is induced by metformin. A**) Rat sciatic nerves (n = 6) were taken 21 days following SNL surgery and ApoE was assessed by Western blot. **B**) Mouse sciatic nerves (n = 6) were taken 21 days following SNI surgery and ApoE was assessed by Western blot. **C**) Mice were treated for 7 days with 200 mg/kg metformin and sciatic nerves were removed and assessed for ApoE expression (n = 6). βIII tubulin was used as a loading control in all studies. ** p < 0.01, *** p < 0.001.

Next, we confirmed our MUDPIT findings with ApoE using Western blot. Comparing ipsilateral to contralateral sciatic nerves from rats 21 days post SNL showed a clear, robust increase in ApoE expression in the sciatic nerve (Figure 3A) as has been shown previously with sciatic nerve crush injury [24]. Furthermore, there is also a robust increase in ApoE expression in the sciatic nerves of mice 21 days after SNI surgery, which suggests that this change occurs across models and across species (Figure 3B). Our proteomic findings also suggest that ApoE is increased in the sciatic nerve after PNI. To assess whether this occurs as a result of metformin treatment in multiple species we treated naïve mice for 7 days with 200 mg/kg metformin and assessed ApoE expression in the sciatic nerve. Metformin treatment led to a doubling of ApoE levels in the sciatic nerve (Figure 3C). Hence, metformin is a legitimate inducer of ApoE expression in the PNS *in vivo*.

## Discussion

The objective of this study was to obtain an unbiased overview of how PNI changes the proteome and functional annotation network of the sciatic nerve and how these changes are further modified by metformin treatment. Here, we report an overview based on MUDPIT and DAVID technology of how pathways are altered in the sciatic nerve distal to PNI and how these pathways are further modified by treatment with metformin. Using this technology, we identified ApoE as a protein profoundly increased and nascently synthesized in the distal sciatic nerve following PNI in rats and mice. Moreover, our results from DAVID analysis reveal that ApoE is a component of a number of functional annotations linked either positively or negatively to metformin treatment that may play a key role in peripheral nerve regeneration and repair or neuronal excitability, respectively. Our results clearly link metformin treatment to further increases in ApoE following PNI and increased expression of ApoE in sciatic nerves of naïve mice. Hence, we conclude that although ApoE has long been linked to PNI, this molecule is a potential regulator of neuropathic pain and/or regeneration following PNI.

Traditional proteomics methodologies separate complex protein samples by isoelectric point and molecular weight in 2-dimensional gels. Patterns are compared between samples by isolating individual protein spots, followed by proteolytic digestion, and analyzing the mass of each peptide by Matrix-assisted laser desorption/ionization-time of flight (MALDI-TOF) mass spectrometry. The measured peptide masses are searched against the predicted mass values for theoretical digestion of proteins in a sequence database, and the protein is identified by a statistically significant number of matches. MUDPIT, which we have utilized here, eliminates gel separations. Instead, biochemical fractions containing many proteins are directly proteolyzed and the enormous number of peptides generated, are separated by 2-dimensional liquid chromatography (repeated cycles of fractionation that extend for 22 hours) before entering the mass spectrometer. Instead of MALDI-TOF, the procedure employs tandem mass spectrometry (MS-MS) so that, after the mass of a peptide is measured, the peptide is fragmented using a collision-induced dissociation cell and the masses of the fragmentation products are determined. Using computational analysis, the data is transformed into an amino acid sequence. Although one peptide is often sufficient to identify a protein using this very sensitive technique, the specificity of this approach improves with increased number of assigned peptides to a particular protein. The advantage of this approach is the identification of minor proteins in a biological fraction that cannot be visualized on 2-dimensional gels. Recent studies have identified 1,000 to 2,000 proteins in a single fraction with MUDPIT [25].

ApoE is a 34 kDa glycoprotein and is a major determinant of lipid transport and metabolism [24]. ApoE is strongly up-regulated after sciatic nerve crush injury where it increases several hundred fold [24] and then declines once regeneration is complete [26]. In this model, it has been proposed that macrophages migrating into the injured site synthesize ApoE, which is then released concurrently with cholesterol and lipids derived from the degenerating myelin as ApoE lipoproteins. ApoE is then taken up by low-density lipoprotein (LDL) receptors on the surface of Schwann cells [27] for recycling and regeneration. However, further studies reveal an important role for ApoE in neuroregeneration and remyelination [24,28,29] suggesting a direct effect of ApoE on neuronal function and, possibly, regeneration. ApoE, for example, is well known as an important mediator of Alzheimer’s disease [30]. ApoE has also emerged as an important regulator of nerve sprouting and nerve regeneration as well as neuroprotection. Several studies have indicated that the addition of ApoE to cultured neurons enhances nerve sprouting [28,31], including enhanced sprouting of dorsal root ganglion (DRG) neurons [28]. After traumatic injury, genetic depletion of ApoE leads to worse outcomes [32-34], whereas enhancing ApoE activity is beneficial [35-40]. More recently, an ApoE mimetic, COG112, was shown to significantly improve recovery of motor and sensory function following sciatic nerve crush in C57BL/6 mice [29]. This indicates a positive modulatory role of ApoE in nerve regeneration. This finding also suggests that further enhancing ApoE expression or action in addition to increases that occur from injury alone are beneficial. This suggests that enhancements observed with metformin may be important in functional recovery after nerve injury. While this hypothesis requires further testing, it is very strongly supported by functional annotation analysis. Finally, ApoE has potent anti-inflammatory properties. Mice lacking ApoE have a substantially exaggerated response to LPS with regard to tumor necrosis factor α (TNFα), interleukin-6 (IL-6), (IL-12) and interferon gamma (IFNγ) [41]. Reconstitution of plasma levels of ApoE in ApoE-knockout mice normalizes LPS-induced IL-12 and significantly reduces LPS-induced TNFα plasma levels. Sustained chronic inflammation is known to be detrimental for functional recovery following PNI [42]. PNI and neuropathic pain are associated with changes in proinflammatory cytokine expression in the PNS, where these factors may play a direct role in sensitizing injured sensory afferents [43]. Thus, stimulating endogenous expression of ApoE, as can be done with metformin administration, may provide benefits by repairing damaged nerves and modulating pain. This effect of metformin may also have important benefits in other neurological pathologies where ApoE might either be deficient or play a beneficial role therapeutically.

There is strong evidence that changes in translation regulation may underlie pathology leading to and maintaining neuropathic pain [5-8,44]. PNI induces a complete reorganization of translational machinery in the PNS [7]. This change is functionally linked to altered sensory processing, mainly allodynia [7] and pin-prick hyperalgesia [5,6], as revealed by behavioral pharmacology studies. One possible drawback of utilizing pharmacological mechanisms to block translation regulation pathways for the treatment of neuropathic pain is a detrimental effect on nerve regeneration due to the key role that translation regulation pathways play in this event, at least *in vitro*[45]. We argue that activating AMPK to achieve regulation of enhanced translation following nerve injury is unlikely to create these adverse consequences. Again, findings using the DAVID algorithm very strongly support this conclusion as they show that metformin-induced increases in ApoE are linked to functional annotations that are predicted to enhance peripheral nerve regeneration and repair. Here several key findings should be considered: 1) while metformin treatment blocks dysregulated translation after PNI, it does not reduce normal translation [7], 2) profiling of the effects of metformin on the translatome reveals that metformin targets only a subset of mRNAs to alter the proteome [46] (consistent with our findings here) and 3) metformin increases ApoE expression which is linked to enhanced functional recovery after PNI. In that regard, it is critical to note that while ApoE participates in a wide range of cellular functions, after metformin treatment, the shift in the proteome changes the context of overall cellular functions such that a set of functional annotations (Figure 2, red bars) containing ApoE and highly enriched in regeneration and repair is revealed. Moreover, metformin treatment reduced functional annotations linked to neuronal excitability induced by SNL (Figure 2, blue bars) consistent with its effect on reducing neuropathic allodynia in rats in this model of neuropathic pain [7]. While we cannot rule out other possible mechanisms, with the exception of AMPK, for these effects of metformin, the safety, clinical availability and tolerability of this drug make it an attractive candidate for human trials for the treatment of neuropathic and possibly other forms of pain [7,47].

## Materials and methods

### Surgery and behavioral testing

Male Sprague Dawley rats (Harlan, 250–300 g) and male ICR mice (Harlan, 18–22 g) were used. All animal procedures were approved by the Institutional Animal Care and Use Committee of The University of Arizona and were in accordance with International Association for the Study of Pain guidelines. Prior to surgery all animals were assessed for mechanical withdrawal thresholds [48]. Spinal nerve ligation (SNL) was done on rats by tight ligation of the L5 and L6 spinal nerves as described by Kim and Chung [49]. Sham control animals underwent the same surgery and handling as the experimental animals but without SNL. Spared nerve injury (SNI) was performed on the mice as described previously [50]. All animals were allowed to recover for 14 days and all testing commenced 14 days post-surgery. Following nerve injury, only animals that developed paw withdrawal thresholds less than 4.7 g for SNL by day 14 were used. Animals were placed in acrylic boxes with wire mesh floors and allowed to habituate for 1 hr. Pre-drug mechanical thresholds were recorded and the animals received intraperitoneal injections of vehicle or metformin (200 mg/kg) [51]. Naïve mice received the same dose of metformin via IP injection. Calibrated von Frey filaments (Stoelting, Wood Dale, IL) were used for mechanical stimulation of the plantar surface of the left hindpaw and withdrawal thresholds were calculated using the up-down method [48]. For Western blotting and proteomic experiments sciatic nerves were harvested on day 21 after PNI.

### Western blotting

Protein was extracted from tissue in lysis buffer (50 mM Tris HCl, 1% Triton X-100, 150 mM NaCl, and 1 mM EDTA at pH 7.4) containing protease and phosphatase inhibitor mixtures (Sigma) with an ultrasonicator on ice, and cleared of cellular debris and nuclei by centrifugation at 14,000 RCF for 15 min at 4°C. Fifteen micrograms of protein per well were loaded and separated by standard 7.5% or 10% SDS-PAGE. Proteins were transferred to Immobilon-P membranes (Millipore) and then blocked with 5% dry milk for 3 h at room temperature. The blots were incubated with primary antibody overnight at 4°C and detected the following day with donkey anti-rabbit antibody conjugated to horseradish peroxidase (Jackson Immunoresearch). Signal was detected by ECL on chemiluminescent films. ApoE was normalized to the expression GAPDH and/or βIII tubulin on the same membrane. Membranes were stripped prior to antibody incubation for normalization. Densitometric analyses were performed with Image J software (NIH). ApoE antibody was from AbCam (ab20874) and used at a 1:2500 dilution.

### Nascent protein synthesis in sciatic nerves

Ipsilateral and contralateral sciatic nerves from SNL rats or ipsilateral sciatic nerves from sham rats were excised at a length of 2 cm. The nerves were then cut to a length of 1 cm and incubated in DMEM/F12 supplemented with 50 μM of Azidohomoalanine (AHA). After 2 hours of incubation at 37°C in a humidified 95% air/5% CO2 incubator, protein was extracted from the nerves by ultrasonication in lysis buffer. AHA incorporating proteins were immobilized on alkyne conjugated agarose resin using Click-iT Protein Enrichment Kit (Invitrogen, Cat # C10416). The click chemistry reaction results in a covalent bond between the azide (AHA) containing nascently synthesized protein and the alkyne conjugated agarose resin which allowed us to eliminate proteins that are not nascently synthesized. The agarose resin conjugated with the nascent proteins was then subjected to trypsin digestion generating the peptides that underwent proteomic analysis.

### Proteomics

We analyzed three samples, each containing 1 microgram of protein pooled from each group (control, injured and metformin) treated by multidimensional protein identification technology (MUDPIT). For these complex protein samples, which were initially prepared in standard Western blot lysis buffer, homogenized and cleared of nuclei and cellular debris by centrifugation as described above, ammonium bicarbonate was added to a concentration of 0.1 M; 40 μL of 10 mM DTT was then added before reducing at 56°C for 45 min. Reduced cysteines were alkylated by addition of 40 μL of 55 mM iodoacetamide (10 mM final concentration) and incubated for 30 min at room temperature. Proteolysis was initiated with a 1:25 ratio (by weight) of sequencing grade modified trypsin and allowed to proceed overnight at 37°C. The digest was stored at −20°C until analysis. For MUDPIT, we used a microbore HPLC system (Paradigm MS4; Michrom, Auburn, CA) with two separate strong cation exchange and reversed phase columns: a 100 μm I.D. capillary packed with 7 cm of 5 μm Vydac C18 reversed phase resin and a separate 250 μm I.D. capillary packed with 7 cm of 5 μm Partisphere strong cation exchanger resin (Whatman, Clifton, NJ). The sample (4.36 μg) was acidified using trifluoroacetic acid (TFA) and manually injected onto the strong cation exchange column, the effluent from the column being fed through reversed phase column. A representative 12-step MUDPIT analysis includes the following solutions: 10% methanol/0.1% formic acid, 0.01% TFA (buffer A); 95% methanol/0.1% formic acid, 0.01% TFA (buffer B); 10% methanol/0.1% formic acid, 0.01% TFA (buffer C); and 500 mM ammonium acetate/10% methanol/0.1% formic acid, 0.01% TFA (buffer D). Step 1 consisted of a 5-min equilibration step at 100% buffer A, another equilibration step for 5 min at 25% buffer B (75% buffer A), and a 40 min gradient from 25% buffer B to 65% buffer B, followed by 10 min 65% buffer B and 10 min 100% buffer A. Chromatography steps 2–12 followed the same pattern: 15 min of the appropriate percentage of buffers C and D followed by a 2-min 100% buffer C wash, a 5-min wash with 100% buffer A, equilibration with 25% buffer B for 5 min, a gradient from 25% buffer B to 65% buffer B in 40 min; and finally a 10-min 65% buffer B wash and a 10-min 100% buffer A wash. The buffer C/buffer D percentages used were 95/5%, 90/10%, 85/15%, 80/20%, 70/30%, 60/40%, 40/60%, 20/80%, 0/100%, 0/100%, and 0/100% for the 11 salt steps. MS/MS and database searching conditions were the same as those described previously [52]. We used scaffold to filter the results according to previous criteria for reliable sequence peptides [53,54]. In scaffold, proteins were identified first by noting assigned peptides for a given protein (sequence match). The number of peptides identified for a given protein were then totaled for all identified proteins. Hence, “assigned peptides” refers to peptides that match a protein based on sequence matching and “number of peptides” refers to the total number of peptides for a given protein identified through MUDPIT. Proteins were assigned to categories for analysis based on the number of peptides identified for the protein according to treatment.

### Gene ontology and functional annotation analysis

We utilized The Database for Annotation, Visualization and Integrated Discovery (DAVID, http://david.abcc.ncifcrf.gov, [55,56]) in order to determine enriched cellular functions following nerve injury or treatment with metformin. The Accession numbers of the list of proteins with at least 1 assigned spectrum from sciatic nerves taken from SNL or SNL+metformin treated rats were submitted as gene list to DAVID. The list of proteins with at least 1 assigned spectrum from uninjured nerve served as the background list. The Functional Annotation Chart was generated using the default annotation categories. The Functional Annotation of ApoE was extracted from the Functional Annotation Table. The data was exported from DAVID and further organized using Microsoft Excel.

## Competing interests

The authors declare no conflicts of interest.

## Authors’ contributions

OKM, HNY and TJP conceived of research plan; OKM, HNY and AS conducted research; OKM, HNY, AS and TJP analyzed data; PKM, HNY, AS and TJP wrote the paper. All authors read and approved the final manuscript.

## Supplementary Material

Additional file 1: Table S1 List of all proteins found in the sciatic nerve by MUDPIT with the number of peptides found in SNL, sham and SNL + metformin listed for each protein. ApoE is in red. **Table S2.** List of all proteins increased by SNL treatment compared to sham animals. **Table S3.** List of all proteins decreased by SNL compared to sham. **Table S4.** List of all proteins increased by SNL + metformin. **Table S5.** List of all proteins decreased by SNL + metformin. Click here for file

Additional file 2: Table S6 List of all GO terms identified in SNL (injured) and SNL + Metformin (Metformin) organized by the number of proteins found per GO term for each treatment. **Graph 1.** The graph shows all the data in Table S6 in graphical form organized by number of proteins per GO term. **Table S7.** All GO terms found in SNL rats with GO terms that contain ApoE shown in red. **Table S8.** All GO terms found in SNL + Metformin rats with GO terms that contain ApoE shown in red. Click here for file

Additional file 3: Table S9 List of all GO terms found in SNL or SNL + Metformin rat sciatic nerves that contain ApoE. **Table S10.** List of all GO terms containing ApoE by number of proteins found in SNL (injured) versus SNL + Metformin (Metformin). Figure 1 and 2. Graphs show Table 10 data in graphical form. Click here for file

Additional file 4: Table S11 Proteins identified from SNL (injured) or sham (control) sciatic nerves that were nascently synthesized, defined by incorporation of AHA. Peptide numbers per protein are listed. ApoE is in red. Click here for file
